# Supplementary material for: Serological and Molecular Survey of Leishmania infantum in a Population of Iberian Lynxes (Lynx pardinus)
Source: Microorganisms. 2022 Dec 11;10(12):2447. doi: 10.3390/microorganisms10122447 (PMC9788222; doi:10.3390/microorganisms10122447)
Supplement: Supplementary file 1 [file microorganisms-10-02447-s001.zip › microorganisms-2044865-supplementary.pdf]

Supplementary Materials

Table S1. Descriptive statistics per each ELISA antigen and cut-off determination. Results obtained from average OD (optical densities) of at least two independent assays performed in technical triplicates. Cut-offs calculated by the average result of 75% quartile + 2x standard deviation (SD).

	SPLA<br>N=36	rK39<br>N=36	CPX<br>N=36	SECA<br>N=36
Minimum	0.004	0.003	0.000	0.004
Maximum	0.073	0.098	0.063	0.076
Median	0.009	0.009	0.010	0.016
25% percentile	0.005	0.006	0.006	0.010
75% percentile	0.013	0.016	0.014	0.030
95% CI of means				
Actual confidence level	97.12%	97.12%	97.12%	97.12%
Lower confidence limit	0.006	0.007	0.007	0.012
Upper confidence limit	0.013	0.015	0.013	0.024
Average	0.012	0.013	0.013	0.022
SD	0.012	0.016	0.012	0.018
SD Error of Mean	0.002	0.003	0.002	0.003

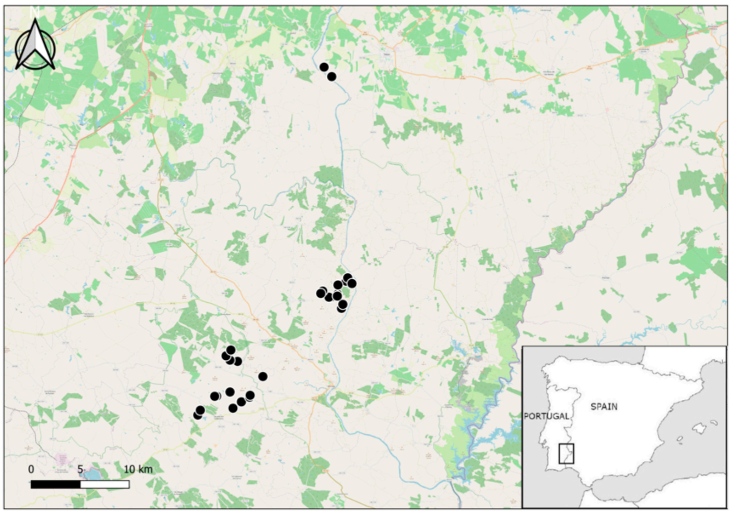

Figure S1. Representative map illustrating capture points alongside the Guadiana River Valley, southeast of the Alentejo region, southern Portugal. Black dots represent location of captured animals.

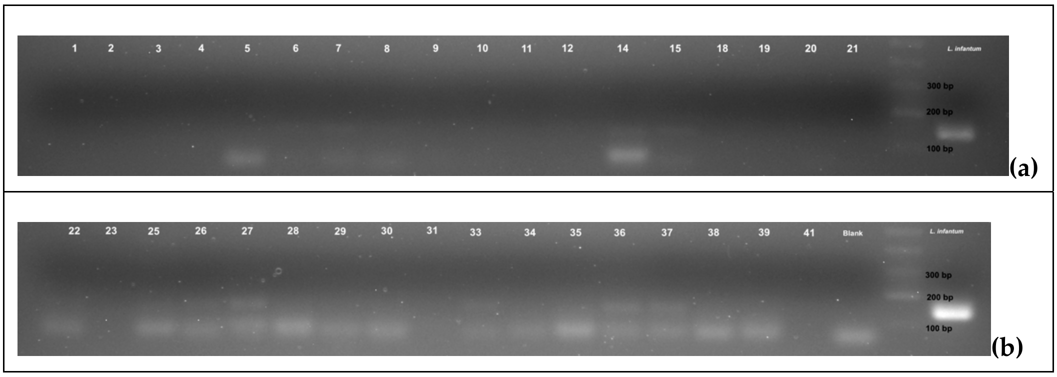

Figure S2. Representative image of *Leishmania* spp. minicircle kDNA detection by PCR (primers RV1 and RV2) on a 1.5% agarose gel electrophoresis, stained with 0.2 μg/mL of nucleic acid stain with a 100 bp molecular marker (Ladder V, NZYTech, Portugal). Each lane is identified by the sample number. *L. infantum* positive control presents a 145 bp band. (**a**) Positive samples: 6, 7, 14 and 15. (**b**) Positive samples: 27, 33, 36, 37.

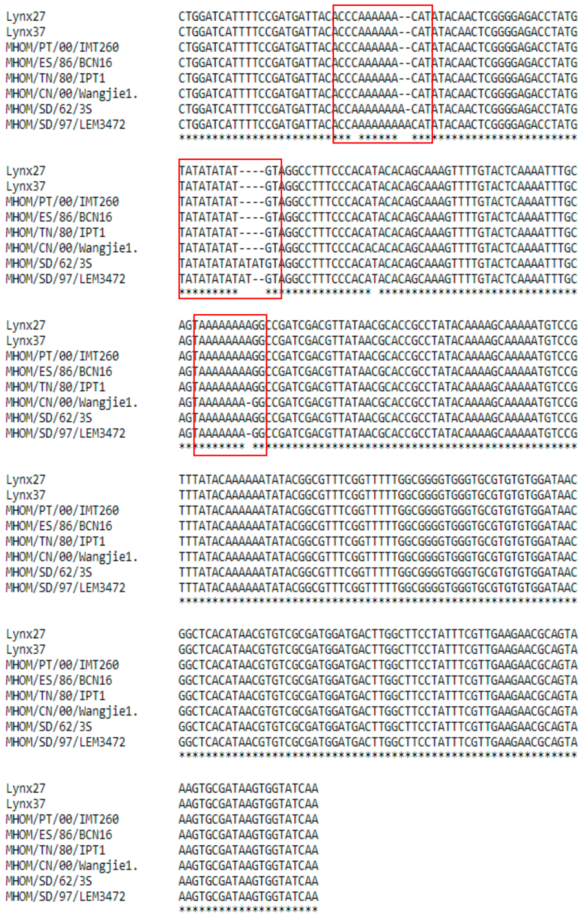

Figure S3. *Leishmania infantum* ITS-1 sequences and main polymorphic regions from samples 27 and 37**.** Multiple sequence alignment was performed using the sequences from Lynxes samples 27, 37 and six other *L. infantum* strains. In red are highlighted polymorphic regions used to define the ITS sequence type.

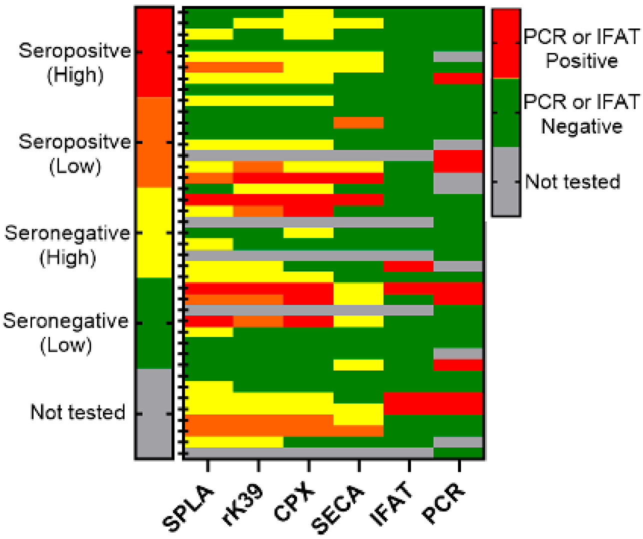

Figure S4. Categorical representation of ELISA seroreactivity against SPLA, rK39, CPX and SECA, in conjugation to PCR and IFAT results. Stratification of ELISA seroreactivity by antigen into "high seropositive" (higher that 1.5x above the cut-off, red color); "low seropositive" (between 1 and 1.5x the cut-off, orange color); "high seronegative"(between 1 and 0.5x the cut-off, yellow color) and "low seronegative"(lower than 0.5x the cut-off, green color).

	SPLA	rK39	CPX	SECA
SPLA		0,88<br>(0.77 to 0.94)	0,73<br>(0.52 to 0.86)	0,49<br>(0.18 to 0.71)
rK39			0,87<br>(0.75 to 0.93)	0,64<br>(0.39 to 0.80)
CPX				0,52<br>(0.22 to 0.73)

Figure S5. Correlation analysis for all samples reporting Spearman's rs values calculated with 95% confidence interval (IC). A very strong correlation is considered for 0.9 > r <1; strong correlation is considered for 0.7 > r <0.89; moderate correlation is considered for 0.4 > r <0.69; weak correlation is considered for 0.4 > r <0.69 and very weak correlation for r < 0.19; confidence interval of 95% is reported in brackets bellow r value.

(a) All Samples

	SPLA	rK39	CPX	SECA
SPLA		1,23E-12	3,89E-07	2,23E-03
rK39			6,82E-12	2,34E-05
CPX				1,10E-03
SECA				

(b) Seropositive

	SPLA	rK39	CPX	SECA
SPLA		3,75E-05	7,89E-03	0,24
rK39			3,10E-03	0,20
CPX				0,59
SECA				

(c) Seronegative

	SPLA	rK39	CPX	SECA
SPLA		5,69E-05	0,08	0,88
rK39			1,77E-04	0,14
CPX				0,73
SECA				

(d) Seronegative (High)

	SPLA	rK39	CPX	SECA
SPLA		2,18E-03	0,509	0,51
rK39			**0,014**	0,07
CPX				0,16
SECA				

(e) Seronegative (Low)

	SPLA	rK39	CPX	SECA
SPLA		0,53	0,81	0,53
rK39			0,12	0,45
CPX				0,91
SECA				

Figure S6. Representation of *p*-values associated to the Spearman's correlation analysis of ELISA seroreactivity reported in Figures 1 and 2.
